# Explanation of factors forming missed nursing care during the COVID-19 pandemic: A qualitative study

**DOI:** 10.3389/fpubh.2023.989458

**Published:** 2023-01-26

**Authors:** Ali Safdari, Maryam Rassouli, Maryam Elahikhah, Hadis Ashrafizadeh, Salman Barasteh, Raana Jafarizadeh, Fatemeh Khademi

**Affiliations:** ^1^Student Research Committee, Baqiyatallah University of Medical Sciences, Tehran, Iran; ^2^Student Research Committee, Hamadan University of Medical Sciences, Hamadan, Iran; ^3^Cancer Research Center, Shahid Beheshti University of Medical Sciences, Tehran, Iran; ^4^Student Research Committee, Faculty of Nursing, Dezful University of Medical Sciences, Dezful, Iran; ^5^Health Management Research Center, Baqiyatallah University of Medical Sciences, Tehran, Iran; ^6^Nursing Faculty, Baqiyatallah University of Medical Sciences, Tehran, Iran; ^7^Department of Medicine, Ardabil Branch, Islamic Azad University, Ardabil, Iran; ^8^Department of Nursing, School of Nursing, Arak University of Medical Sciences, Arak, Iran

**Keywords:** missed nursing care, qualitative study, COVID-19, pandemic, nursing, Iran, hospital, quality of care

## Abstract

**Background:**

Providing nursing care to patients with COVID-19 has put additional pressure on nurses, making it challenging to meet several care requirements. This situation has caused parts of nursing care to be missed, potentially reducing the quality of nursing care and threatening patient safety. Therefore, the present study aimed at explaining the factors forming missed nursing care during the COVID-19 pandemic from the perspective of nurses.

**Methods:**

This qualitative study was conducted using a conventional content analysis approach in Iran, 2020–2021. Data were collected from in-depth, semi-structured interviews with 14 nurses based on purposive sampling. Data analysis was performed simultaneously with data collection. Graneheim and Lundman's approach was used for data analysis, and MAXQDA software was used for data management. After transcribing the recorded interviews, to achieve the accuracy and validity of the study, the criteria proposed by Lincoln and Guba were considered and used.

**Results:**

A total of 14 nurses with a mean age and standard deviation of 31.85 ± 4.95 and working in the COVID-19 wards participated in the study. The acquired data were categorized into four main categories: care-related factors, disease-related factors, patient-related factors, and organization-related factors. The category “care-related factors” comprised uncertainty in care, PPE-related limitations, attrition from care, and futile care. The category “disease-related factors” consisted of the extension of symptoms, unpredictable peaks of the disease, and restriction on the presence of patients' companions. The category “patient-related factors” included comorbidities, elderly patients, and deterioration of infected patients. Ultimately, the category “organization-related factors” consisted of restrictions on equipment supply, lack of human resources, weaknesses in teamwork, and an unsupportive work environment.

**Conclusion:**

The results of this study showed that several reasons including factors related to care, patient, disease, and organization cause missed nursing care. By modifying the related affecting factors and considering the effective mechanisms to minimize missed nursing care, it is possible to provide better services.

## 1. Introduction

To date, the COVID-19 pandemic has been one of the biggest public health crises (1). According to a recent report by the World Health Organization (WHO), 22 December 2022–to date, about 6,671,610 people have lost their lives to COVID-19 (2). The health systems of different countries have been subjected to extensive pressure and restrictions due to the severity, volume, extent, and uncertainty in the treatment of the COVID-19 pandemic (3). Furthermore, the lack of sufficient financial resources and personnel has played an outstanding role in aggravating these conditions to deal with this health crisis (4). Nurses caring for patients with COVID-19 have faced many other challenges, including working in a new environment, being worn out by the workload, the struggle of wearing protective gear, fear of COVID-19, witnessing suffering, keeping their patients safe with extra measures, teamwork, and a true calling (5). Moreover, facing the current critical situation, health workers and nurses are at risk of experiencing psychological distress including anxiety, fear, depression, and insomnia (6). Recent studies indicate that nurses are unable to meet the complex care needs of patients with COVID-19 and feel helpless to perform their duties (7, 8). These conditions have led to the loss of an essential part of the care of patients with COVID-19 (9).

Missed nursing care includes any clinical, administrative, or psychological care that is (in whole or part) delayed or not performed during a given shift (10). Missed nursing care, also known as implicitly rationed care, incomplete care, or unfinished care, is a prominent concern in many healthcare systems worldwide (11, 12). Smith et al. (13) stated that 49% of nurses in the United States admit to missing at least one or more nursing care tasks. Meanwhile, Cho et al. stated that 81% of nurses in Korea admit to missing nursing care (14). When the number of patients the nurse cares for reaches 7–11, the percentage of missed nursing care increases up to 89% (15). Nilasari et al. (16) described observations in a hospital, where nurses neglected to provide conditions, nutrition, and personal hygiene. According to the global prevalence rates ranging from 55 to 98% (17), missed nursing care often occurs when nurses do not perform required nursing care tasks due to the enhancement of patient care demands, insufficient manpower and financial resources, or other challenges. Missed nursing care is associated with negative outcomes for patients, nurses, and the organization (18), which has negative consequences such as increased medication errors, pressure ulcers, infection, falls, mortality, and patient dissatisfaction (19).

Factors that caused missed nursing care vary in different countries depending on the work environment, available resources (human and financial resources), cultural factors, and the existence of specific guidelines or protocols in each country (20). The nursing work environment such as very few staff, the time required for the nursing intervention, poor use of existing staff resources, and ineffective delegation largely predicts the amount of missed care and the factors influencing its formation (21). On the contrary, fulfilling all care requirements in a pandemic is improbable (22), and missed nursing care is inevitable and unpredictable (23). The consequences of missed nursing care in a recent study in Iran during the COVID-19 pandemic were described as follows: “moral distress,” “job dissatisfaction,” “reduced quality of nursing care,” “patient dissatisfaction,” “adverse effects,” “absence of work,” “leave intention and subsequent turnover,” “reduction of hospital credit,” and “increase of hospital costs” (24, 25). Safdari et al. in a qualitative study reported that the causes of missed nursing care were categorized into four categories, namely, “unfulfilled care,” “care at an improper time,” “incomplete care,” and “incorrect care” (25).

The COVID-19 pandemic required new methods of recruiting staff, transferring nurses to other units, and forcing them to work in new roles and tasks as well as working with new colleagues. The number of patients was expected to increase, and there was insufficient knowledge about how to care for patients with COVID-19. These conditions can potentially affect the quality of care and patient safety (26).

To study human phenomena, the evaluation of different perspectives is the best qualitative method. Humanity, social, cultural, and relationship dimensions and values cannot be described well through quantitative approaches (27). By using qualitative study, it is possible to discover, describe, and explain unknown or little-known phenomena from the language of those who experience those phenomena in different cultures (28). Due to the fact that missed nursing care has recently raised wide concerns in the field of health and treatment, and despite its high prevalence and importance, few studies have been conducted on it. The effort to prevent missed nursing care needs extensive research in the field of health and treatment (29). Since the coronavirus pandemic, the world is facing a huge amount of missed care in addition to reported medical errors, and this phenomenon has received less attention and focus in Iran, so there is little information about this concept, the extent of this problem, and the factors related to it. Patient care experiences during a pandemic can be useful for optimizing patient care delivery in the future and current crises (30). Therefore, the present qualitative study was conducted with the aim of explaining factors forming missed nursing care during the COVID-19 pandemic from the perspective of nurses.

## 2. Materials and methods

### 2.1. Design and setting

The present qualitative study was conducted using a conventional content analysis approach (31). This study was conducted from December 2020 to February 2021 in Tehran. The selected hospitals as the study setting included Baqiyatallah Al-Azam in Tehran and Amir Al-Momenin and Ayatollah Khansari in Arak. These three hospitals were considered referral centers for patients with COVID-19. The study report was presented using the consolidated criteria for qualitative reporting research (COREQ) (32).

### 2.2. Participants and sampling

We considered at least 2 years of nursing experience as well as 1 month of caring for patients with COVID-19 as sufficient experience. Demographic and clinical variables are given in Table 1. The inclusion criteria included having formal care experiences of the patient with COVID-19 and having experience of missed nursing care in caring for patients with COVID-19. After the agreement, no participants refused or withdrew. Participants were purposefully selected with maximum variation (selecting nurses from different parts of the country and different hospitals who can represent the nursing community). Purposive sampling, also known as judgmental, selective, or subjective sampling, is a form of non-probability sampling in which researchers rely on their judgment when selecting members of the population to participate in their surveys. This sample is selected in such a way that it has the characteristics of real society as much as possible. Finally, 14 nurses were interviewed as participants in the study. Interviews were conducted after obtaining the consent of the participants. The place and time of interviews were arranged based on the participant's preference and agreement to ensure their comfort during the interview. Interviews were conducted by AS, a 32-year-old male nursing graduate student with 5 years of nursing experience. Before the interview, he had participated in a qualitative study course and had received training in a qualitative study, and interviewing, coding, and reporting were done by SB. Data collection was carried out until saturation, and finally, no new information was added after 14 interviews. The saturation point is reached when no new evidence is obtained from the data. In other words, complete verification of the data has been done and no new category has been created.

**Table 1:** Demographic and clinical information of study participants.

**Participant no**	**Age (year)**	**Sex**	**Experience (years)**	**Ward**	**Shift circulating**	**Education level**	**Duration of interview (minutes)**
P1	30	Female	6	General	Rotation	Bachelor	50
P2	28	Female	5	General	Morning/ evening	Bachelor	56
P3	35	Female	10	ICU	Rotation	Masters	45
P4	29	Female	4	ICU	Rotation	Masters	46
P5	29	Male	5	Emergency	Rotation	Masters	56
P6	33	Female	8	General	Night	Masters	52
P7	39	Male	15	Emergency	Rotation	Masters	63
P8	32	Female	7	General	Rotation	Bachelor	45
P9	28	Female	7	General	Rotation	Bachelor	56
P10	33	Female	10	General	Morning	Bachelor	50
P11	44	Female	18	ICU	Rotation	Bachelor	52
P12	33	Female	8	General	Rotation	Bachelor	45
P13	27	Female	3	General	Rotation	Bachelor	52
P14	26	Male	2	Emergency	Rotation	Bachelor	60

### 2.3. Data collection

In-depth and semi-structured interviews with participants were used to collect data. Data collection and analysis were done simultaneously. Each interview was conducted by the first author. In-depth semi-structured interviews evoke very meaningful narratives. These interviews turn questions on a specific topic into storytelling invitations (33). This type of interview is perceived as ‘talking' and talking is natural (34). The average interview time was 52 ± 5.71 min and was continued until data saturation was met. The interviews continued until data saturation was reached; in other words, no further codes or subcategories were achieved. In other words, we achieve data saturation (35). The interview was organized in four phases (36). They are (1) the orientation phase: the researcher introduced himself and the title of the study, and permission to record the interviews was obtained; (2) the main question phase: the main question was asked; (3) the probing phase: depending on the experiences of participation, further questions were asked; and (4) the final phase: at the end, participants were allowed to add any further information if they were willing. The details of questions are presented in Table 2.

**Table 2:** The interview questions.

	**Questions**
Primary questions	Mention “your experience of caring for COVID-19 patients”
	The next questions are based on the participants' experiences
	Examples:
	What were the conditions of COVID-19 patients?
	- How many covid-19 patients did you care for daily? - What stress did you experience while caring for these patients? - What was the intensity of the shifts? - How was your experience with the high number of deaths of COVID-19 patients?
Main question	What were the reasons for the missing of nursing care during the COVID-19 pandemic?
Probing questions	Based on the participants' experiences probing questions were asked. Examples:
	- What care is missed while caring for COVID-19 patients? - How much of your care was missed during this pandemic? - What factors contributed to the missing of nursing care during the COVID-19 pandemic? - How and under what circumstances was this care missed?
Terminal question	Do you have any other points or questions?

### 2.4. Data analysis

To analyze qualitative data, Graneheim and Lundman method was used (37). For encoding and data management, MAXQDA software version 10 was used (38). The coding process was conducted by removing duplicate codes and merging similar codes according to constant comparative analysis. The interviews were transcribed by AS. AS and SB coded the interviews and they read them several times to gain an understanding of the entire interview. Following that, we identified the meaning units and the primary codes. The text was divided into semantic units that were compressed. The compressed meaning units were abstracted and labeled with a code. Afterward, similar primary codes were condensed and merged into subcategories, and the main categories were extracted. Qualitative data analysis based on the method proposed by Graneheim and Lundman is presented in Table 3. An example of data analysis is presented in Table 4.

**Table 3:** Qualitative data analysis process based on Graneheim and Lundman method (33).

**Phase**	**Method**
Creating content in text format	Transcribing the entire interview immediately after each interview
Getting a general sense	The text of the interview was read several times by the researcher.
Creating initial codes	Determining the meaning units and labeling by the initial codes
Creating subcategories	Classifying similar primary codes into more comprehensive subcategories by grouping the number of codes and merging similar codes based on their differences and similarities
Creating categories	Reducing the number of subcategories and merging them into more general units based on conceptual and logical relationships with other categories

**Table 4:** An example of data analysis.

**Main categories**	**Subcategories**	**Basic codes**	**Quotation**
disease-related factors	Unpredictable peaks of the disease	Shortages caused by the start of new peak	“*At the onset of the pandemic, when the number of hospitalizations was high, we faced great shortages. Now, when the number of hospital admissions increases unpredictably, we get into trouble every time, and we can't take care of the patients as we should and had to eliminate a lot of care...” (P4)*

### 2.5. Trustworthiness

By considering credibility, transferability, dependability, and confirmability as Guba and Lincoln's criteria, trustworthiness was improved (39). Based on this, the credibility of the data was determined through a prolonged engagement with the participants, returning the interview text to the participants and obtaining their approval, and evaluating the transcripts, codes, and themes of the interviews by three experts. In addition, reliability was determined using a combination of methods for data collection (interview, observation, and field notes). We spend enough time collecting and immersing in the data from December 2020 to February 2021. Besides, to conduct member check and peer check techniques, the findings were checked by some participants and two other researchers, respectively. To achieve dependability and confirmability, the research team attempted to achieve a definitive organization of data (all research processes, particularly data collection, data analysis, and formation of categories, were recorded and presented to be used by audiences and readers); consequently, a common consensus was reached. For transferability, a detailed analytical description of the context, methodology, and limitations was reported to an external observer who was familiar with qualitative studies. Also, maximum variation sampling was done by interviewing nurses with different education, wards and shifts, and different ages.

## 3. Findings

A total of 14 nurses (14 interviews were conducted) with mean age and standard deviation of 31.85 ± 4.95 working in the COVID-19 wards participated in the study. The participants' characteristics are presented in Table 1. A total of 310 codes were extracted and 63 duplicate codes were found. Factors affecting the formation of missed care during the COVID-19 pandemic emerged in four categories and 14 subcategories (Table 5). The categories were “care-related factors,” “disease-related factors,” “patient-related factors,” and “organization-related factors.”

**Table 5:** Factors influencing missed nursing care.

**Category**	**Subcategory**
Care-related factors	Uncertainty in care
	PPE-related limitations
	Attrition from care
	Futile care
Disease-related factors	The extension of symptoms
	Unpredictable peaks of the disease
	Restriction on the presence of patients' companions
Patient-related factors	Comorbidities
	Elderly patients
	Deterioration of infected patients
Organization-related factors	Restrictions on equipment supply
	Lack of human resources
	Weaknesses in teamwork
	Unsupportive work environment

### 3.1. Care-related factors

The limitations and complexities of caring for patients with COVID-19 lead to numerous interruptions in the treatment process, wasting time, and sometimes hasty or delayed nursing decisions. This category consists of four subcategories: “Uncertainty in care,” “Personal Protective Equipment (PPE)-related limitations,” “Attrition from care,” and “Futile care.”

#### 3.1.1. Uncertainty in care

Some participants mentioned that the confusion caused among nurses due to constant changes in nursing guidelines designed to identify and care for patients with COVID-19 had led to uncertainty and ambiguity in the provision of nursing services. Despite over a year since the onset of the pandemic and accumulated clinical experiences, participants believed that the disease was still not fully known and that there were many unknowns regarding the prevention, treatment, and care of patients among healthcare providers that had caused existing treatments to be ineffective.

“*... According to what we were told, we used to think that it would be better to place our patients in a semi-sitting position in order to help them breathe better. We had one or two patients who had a lowering in blood pressure when we used an NIV mask for them, and their oxygen saturation level was still low” (P13)*.

#### 3.1.2. PPE-related limitations

Continued use of personal protective equipment (PPE) had caused numerous problems for nurses, including reduced mobility and agility, wasting time on wearing and using them, fatigue, sweating and heat, difficulty in physical distancing and having contact with patients, and the inability of patients to recognize nurses' sex. In addition to creating fear of insufficient factors in dealing with patients, PPE has also reduced the frequency of nurses' contact with patients.

“*At the start of each shift, they first give us a mask and a gown, and we have to use it until the end of the shift. If it gets infected, they don't give us an extra one... We sweat a lot under these clothes; it's really hard, we can't talk to and contact patients properly. We can't do much of the patient's work properly under this situation” (P12)*.

#### 3.1.3. Attrition from care

Conflicts between fear and conscience and between fatigue and commitment due to the constant exposure of patients with an infectious disease, high patient mortality, and continued use of PPE have led to attrition from care. Attrition from care leads to psychological problems (such as depression and anxiety) in nurses, threatens the continuation of nursing care, and ultimately leads to the elimination of part of nursing care.

“*We sometimes got into moral conflicts. We were both afraid of being exposed to patients' discharge and we felt responsible.... I remember that in one shift four of our patients died.... This personal protective equipment was added to all the stories…” (P8)*.

#### 3.1.4. Futile care

It is difficult for a nurse to perform futile care because despite knowing that an action is futile, he/she had to do it, and this issue causes moral conflicts, resulting in unwillingness to work, especially in the special care department. In the meantime, the high mortality rate of patients with COVID-19 and the death of young patients has led to the care interventions, not only would they not improve patient prognosis, health, comfort, and wellbeing but they would also lead to a waste of nurses' time and lack of sufficient opportunity to provide basic care to patients, resulting in the omission or neglect of some care.

“*... When we really know that the patient's condition is very complicated, she/he has a very severe disease and she/he will not return to life and they are waiting for her to expire, it means that our care is in futile” (P6)*.

### 3.2. Disease-related factors

The unpredictable nature of COVID-19 has led to various disease peaks in the community and the influx of large numbers of patients with a wide range of symptoms into hospitals. This category consists of three subcategories: “Extension of symptoms,” “Unpredictable peaks of the disease,” and “Restriction on the presence of patients' companions.”

#### 3.2.1. Extension of symptoms

The complexity caused by the nature of corona disease, the severity of the disease, extensive and systematic side effects, the multitude of drugs used to treat the disease, and the mechanisms of the drug's effect have created many tasks for nurses, which lack knowledge and awareness in most cases, and finally, this issue increases the workload of nurses. Ultimately, this has caused nurses to omit some essential care.

“*Different classes of drugs such as respiratory, gastrointestinal, cardiac, etc., are used for patients…. We suddenly came across too many drugs such as Tocilizumab, Adalimumab, Remdesivir… We did not know the mechanism of action and even the side effects of many of them…There are a lot of tasks related to drug administration and the patients' tasks, most of which remain uncompleted at the end of the shift” (P9)*.

#### 3.2.2. Unpredictable peaks of the disease

The sudden increase in new cases of the disease leads to the loss of balance between demand and facilities and staff, and subsequently not providing full nursing care to patients.

“*At the onset of the pandemic, when the number of hospitalizations was high, we faced great shortages. Now, when the number of hospital admissions increases unpredictably, we get into trouble every time, and we can't take care of the patients as we should and had to eliminate a lot of care...” (P4)*.

#### 3.2.3. Restriction on the presence of patients' companions

Participants believed that the impossibility of companions' presence at the patients' bedsides created psychological problems for patients and their families and made it difficult for families to meet patients' basic needs.

“*Most patients have no companions; that's why they call the nurse all the time. Because we don't have any nursing -assistant, we don't have the opportunity to respond to all the patients' requests, so some of our patients get upset” (P3)*.

### 3.3. Patient-related factors

Diverse sociocultural status, combined psychosocial problems, comorbidities, elderly patients, and deterioration of patients have led to a lack of maximum care and the missing part of care.

#### 3.3.1. Comorbidities

Nurses somehow deal with the complexity and confusion of caregivers due to the inability to provide correct and quality care due to the lack of access to reliable and valid information and the lack of proper understanding of the received information, as well as the lack of previous exposure to the care of chronic patients on the one hand and the unpredictable nature of the disease and its consequences on the other hand. The presence of comorbidities in some hospitalized patients and the need to provide related care had led to an increase in nurses' workload and sometimes the elimination of some essential care.

“*Our colleagues are under pressure. Imagine a patient undergoing dialysis who has been admitted to the ward. What problem do you think we should address? We should be alert not to miss the potassium and urea tests of the patient and address the respiratory problem at the same time” (P13)*.

#### 3.3.2. Elderly patients

Commitment and compliance with the ethical principles of care is an important task in nursing practice, which takes precedence over care in providing care to the patient. Therefore, compliance with professional ethical standards in the nursing care of patients is considered very important and necessary. According to the evidence and for various reasons, our nurses are not taking care of the patients and our culture in an ideal way, and there has been negligence toward this issue. For example, more than half of the hospitalized patients are elderly and experienced much higher disease severity than other patients. On the contrary, in addition to the need for COVID-19-specific care, these patients also needed elderly care. Nurses inevitably prioritized and addressed patients' critical care needs. This had led to the delay or elimination of some patient care and services.

“*The conditions of some COVID-19 patients, especially very old ones, are very critical and serious. Some of them were coded together in a single shift, and we had CPR consecutively. I was so busy that I couldn't monitor the vital signs of my patients in that shift” (P4)*.

#### 3.3.3. Deterioration of infected patients

During the peak of this pandemic, many critically ill patients were admitted to the intensive care unit. Due to the weakness of management at different levels, the admission of a large number of patients in need of vital services, more equipment, specialist nurses, and organizational management increased significantly. Therefore, due to the deterioration of the condition of the affected patients, part of the service was lost.

“*Our ward used to be CCU. We generally took care of heart disease. But now, our ward has become COVID ward. Now we have a large number of critically ill patients whose type of care has changed. We no longer do those previous things. We must provide ICU care to the patient. We don't take care of ourselves on our own. It is not in our hands” (P6)*.

### 3.4. Organization-related factors

Weaknesses in the formulation and implementation of national and organizational policies in the absence of explicit guidelines, changes in the use and staff of different inpatient wards without prior planning due to the need for immediate decisions to manage the current crisis, organizational constraints in providing adequate resources (human and financial), and lack of job and social support for nurses are among the factors influencing the elimination of nursing care. This category consists of four subcategories: “Restrictions on equipment supply,” “Lack of staffing,” “Weakness in the team and inter-professional cooperation,” and “Unsupportive work environment.”

#### 3.4.1. Restrictions on equipment supply

Lack of equipment and supplies following the increase in demands had led to delays and the elimination of some patient care.

“*You may not believe that we also have a shortage of suction catheters in the hospital! We have to work in this situation. But, the shortage of equipment sometimes interferes with the treatment and care. It was only yesterday that the buttons of one of the ventilators no longer worked. We barely kept the patient alive with a mask and a bag valve mask until a new ventilator was attached to him” (P10)*.

#### 3.4.2. Lack of human resources

Restrictions on the employment of professional nurses have led to the employment of nursing students, staff passing their training courses, retirees, and nurses with no work experience in infectious disease wards and intensive care units.

“*Our managers sent all the* staff passing their training course *and novice staff to our ward. You know, there're a lot of tasks associated with COVID-19 patients. Our staff can't even find a vein. They aren't to blame; they've just entered this profession... They aren't fast enough. We see the drugs aren't administrated even until the end of shift” (P5)*.

#### 3.4.3. Weaknesses in teamwork

The change in the composition of the care team following the change in the previous wards, the dispatch of nurses to the infectious disease centers, and the recruitment of a large number of new and non-specialist staff have led to changes in professional relationships and disagreements over providing patient care. This weakness in inter-professional cooperation and the care needs of patients had led to the elimination of some nursing care.

“*In our ward, the physicians don't care much about us and the care we provided. They don't listen to us when we talk about the patient, and they do their job” (P6)*.

#### 3.4.4. Unsupportive work environment

Expectations beyond nurses' capacity, inequality, and discrimination in salaries and bonuses to nurses who are at the forefront of the fight against COVID-19 and the lack of power and autonomy of nurses in the healthcare system as professional personnel had reduced nurses' organizational commitment.

“*We've been in the COVID-19 ward for several months and in contact with the patients throughout the shifts, and in constant danger. But when they want to prioritize the staff's hard work, the nurses are the last priorities ... Well, this discrimination affects our motivation to work, and some staff may not work as they should do it” (P11)*.

## 4. Discussion

Studies show that the current pandemic has affected the process of providing care to patients (3, 28, 30). Nurses at the frontline of dealing with this disease are facing challenges in providing appropriate care to patients. Therefore, the present study aimed to explain the factors forming missed nursing care during the COVID-19 pandemic from the perspective of nurses. In their view, meeting all care requirements in a pandemic is highly challenging, and nurses have to rationalize, delay, or eliminate nursing care as missed nursing care (MNC). Participants in this study described the factors affecting the formation of missed care during the COVID-19 pandemic in four domains, namely, care-related factors, disease-related factors, patient-related factors, and organization-related factors.

“Care-related factors” were among the main categories extracted from the interviews which consist of five sub-classes, which are as follows: “Uncertainty in care,” “PPE-related limitations,” “Attrition from care,” “Futile care,” and “Restriction on the presence of patients' companions.”

Most participants stated that confusion in the face of uncertainty, ambiguity, and continuous changes in care protocols of COVID-19 was an important factor leading to MNC. The experience of previous pandemics likewise showed that the initial planning for patient care was not feasible due to the unpredictability of the conditions during the pandemic (40). Tan et al. expressed unfamiliarity with the environment and disease as one of the negative experiences of frontline nurses against COVID-19 (41). Furthermore, uncertainty about care in the presence of factors such as a high number of dying patients and the lack of relationship between the severity of symptoms and the death of patients had led to nurses' perception of futile care and consequently their negligence in performing some tasks and reduced quality and quantity of nursing care. This factor is consistent with the concept of “understanding incompetence in rescuing patients with COVID-19” in Sheng's study (8).

Nurses argued that constant use of PPE had led to reduced mobility, wasted time, and fatigue. In addition, PPE was influential in establishing communication with patients due to patient's inability to recognize the gender of nurses and create an intimate and trustworthy environment. The combination of these factors led to interruptions in the provision of care. Similarly, Ferrari et al.'s study noted the effects of PPE on the trustworthiness relationship, its impact on communication resources, and strategies for overcoming communication barriers as PPE-related limitations (42). Also, Hoernke et al. noted that care continued to be provided despite the physical discomfort, practical problems, and communication barriers associated with PPE use (43).

Participants stated that the conflicts between fear and conscience and between fatigue and commitment in the constant exposure to patients with the infectious disease were among the factors causing attrition from care followed by an inability to provide appropriate care to patients. Other studies have shown that nurses find it challenging and sometimes impossible to meet all care requirements in the face of job stress, and they may shorten, delay, or eliminate some of the necessary care (44). Galehdar et al. also mentioned that caring for patients with COVID-19 affects all aspects of nurses' work and life including the bad feeling of inefficiency, problems of providing care and pollution, a prisoner in the fence of protective equipment, and workload. This can lead to the disintegration and gradual erosion of patient care over time (45). Gao et al. in China stated that nurses' long working hours during the current pandemic were a compelling factor in increasing errors and adverse outcomes of the disease and reducing occupational performance and the quality of nursing care (46). Therefore, it is suggested that by improving the working status of nurses while caring for patients with COVID-19, the attrition from care can be directly or indirectly reduced.

Futile care is another dimension of missed care. Sometimes failure to treat patients leads to a sense of the futility of care (47). A total of 73.80% of the nurses in the study by Eftekhar Ardebili et al. believed that the care they provided to patients with COVID-19 was almost futile, especially in cases where patients died despite all their interventions (48). In this situation, nurses suffer from a mismatch between their best efforts to save the patient and the feedback they receive from the patient (8). In addition, differences in the role of physicians and nurses in understanding the nature and examples of futile care have led to a gap in their perception of this concept (49).

Nurses also have an alternative role of caring for the patients' families due to the restrictive guidelines on the presence of companions at patients' bedsides (50). Nurses stated that patients were dissatisfied with restrictions, which, in addition to creating psychological problems associated with the disease, had caused serious challenges for them in adherence to treatment and participation in care. However, Dehghan Nayeri et al. mentioned that family and companions' presence at patients' bedsides in Iranian hospitals caused congestion in patients' rooms and delays in providing emergency services. Families also caused some care to be missed by raising numerous questions, performing some care arbitrarily, and interfering with nursing care provision (51). This difference can be due to issues such as social isolation and widespread stigma toward patients with COVID-19, and consequently the need to accept them as a person in need of care similar to other diseases and more emotional and psychological support from the family.

The second category from this study was disease-related factors, which was divided into two subcategories (The extension of symptoms and Unpredictable peaks of the disease). Due to the severity of symptoms in some patients, nurses were forced to prioritize and address their critical care needs. There has always been this concern that paying more attention to patients' vital situations prevents nurses from fulfilling the basic care needs of other patients (10). It seems more likely to miss some care in this situation. Therefore, it is essential to educate nurses to make decisions, prioritize, and classify patients in need of critical care.

Due to the disease's wide range of symptoms and complications in patients, the fact that it is not limited to respiratory problems, its impact on various body systems, and underlying diseases, patients need a variety of medications and care, resulting in an increased workload of nurses. It, in turn, requires necessary knowledge about the application, complications, care, and appropriate nursing training. Unexpected increases in nurses' workload and the occurrence of urgent conditions for patients are the main causes of MNC (23). The results of this study are consistent with a similar study before the pandemic in Iran, which considers factors such as the patient's condition and the type and nature of the disease to be effective in the miss of nursing care (51). Similarly, in a study of Crowe et al. conducted during COVID-19, nurses mentioned the factors were due to the changes in patient management methods and restrictions (e.g., limited patient contact) and not being able to provide centered nursing care to prevent the spread of the virus (7). Danielis and Mattiussi also argued that due to the nature of COVID-19, nurses were forced to prioritize nursing care tasks that address patients' oxygenation status positioning to maximize lung expansion, and administration of antibiotics and antiviral drugs more than other nursing care tasks such as maintaining personal hygiene, nursing supervision, and other communication and interaction with patients (52).

The third category from this study was patient-related factors, which were divided into three subcategories (comorbidities, elderly patients, and deterioration of infected patients). Due to special conditions such as complex care, comorbidities, and disability, aging is mainly accompanied by missing parts of care. Fitzgerald argues that in the complex and changeable hospital environment, nurses mainly prioritize the necessary care to be implemented first, left undone, or missed (53). Moreover, unexpected and unforeseen increases in new patients' admission rate following the new waves of infection caused imbalance and instability in the number of facilities and workforce between the two pandemic peaks, each time leading to the imposition of widespread pressure on healthcare providers. Unexpected increases in the admission and discharge of patients and the existence of emergencies led to the failure to provide some care for patients (49). On the contrary, efforts to shorten the hospital stay length to admit new patients and the tendency to outpatient care led to a moderate level of accuracy during hospitalization (54).

The final category from this study was organization-related factors, which were divided into four subcategories (restrictions on equipment supply, lack of human resources, weaknesses in teamwork, and unsupportive work environment).

Our study quotations revealed that the lack of equipment, supplies, and intensive care beds was out of nurses' control and authority, which inevitably caused some care to be missed. In the study by Dehghan Nayeri et al., likewise, issues such as wasting nurses' time due to the use of old, defective equipment and the lack of facilities and equipment were mentioned as factors affecting MNC in health centers in Iran (55).

Another issue argued by participants was the insufficient number of nurses to care for patients infected with COVID-19. The WHO acknowledges a shortage of approximately 6 million nurses worldwide (55). The shortage of employed nurses is considered a significant challenge facing the health system in Iran (56). Statistics show that in Iran, there are 1.97 nurses per 1,000 population (57). In contrast, in China, the number of nurses is only 2.73, United States (2014) 8.76, Japan (2015) 11.46, and Germany (2015) 13.51 per 1,000 people (58), which indicates a significant shortage of nurses to provide optimum care in Iran. Similarly, in the study by Tan et al., the shortage of nurses significantly affected the quality of nursing care and the provision of timely care (41). Having a sufficient number of professionally qualified and experienced nurses increases efficiency, has positive outcomes in disease control, and reduces patient mortality (59).

Another challenge faced by nurses is the ambiguity of roles and responsibilities as vague definitions of jobs, expectations from them, and their responsibilities (60). Nurses mentioned that the lack of physicians' attention to their professional role in patient care could result in missed nursing care. In the same vein, the study by Albsoul et al. showed that poor communication between the nursing team and medical staff was an important factor in MNC (23). However, it is expected that, by transferring a significant number of healthcare workers to new wards, the weakness in teamwork following the pandemic may lead to the formation of new and unfamiliar teams in in-patient centers, aggravating the situation (61).

Nurses also emphasized that discrimination in salaries, poor support in recognizing and participating in decision-making, and low motivation to care for patients in crisis were some of their issues in MNCs. Sheng et al. mentioned that “unexpected professional benefits” were part of Chinese nurses' experience at the forefront of the fight against the virus. Nurses participating in this study felt unfair due to the imbalance between their work and rewards and benefits compared to other healthcare providers, including physicians, which implied ignoring their professional role in fighting the virus (8). Fernandez et al. also argued that nurses need governments, policymaking, and nursing groups to act in supporting nurses, both during and after a pandemic or epidemic (62).

Missed nursing care is a key indicator of patient safety and the evaluation of the quality of nursing services. By identifying factors associated with MNC, appropriate interventions can be administrated (61). Therefore, explaining the factors affecting MNC in different clinical health conditions leads to comparable standard methods and improves the quality of nursing care and patient safety by providing practical solutions to eliminate or reduce this problem (10, 23). However, the frequency and type of MNC in different countries depend on the amount and distribution of resources, cultural and social factors, as well as other variables in the nurses' work environment (20, 49).

## 5. Conclusion

The results of this research showed that from the perspective of the participants in the research, the most important effective factors in missing nursing care are categorized into four themes, namely factors related to care, factors related to the patient, factors related to the disease, and factors related to the organization. The results of this study show that an unpredictable, varying, and stressful work environment, limited human and financial resources, and special characteristics of the disease and infected patients are major challenges for nurses in providing adequate, effective, efficient, and timely care. Therefore, it is recommended to minimize MNC by reviewing care policies and strategies in accordance with the current critical situation, adopting management strategies such as providing sufficient human resources and increasing nurses' job satisfaction, developing and using instruments for measuring MNC according to the Iranian culture and context, strengthening quantitative research according to qualitative studies conducted in Iran, and sensitizing of nursing managers to adjust the MNC to increase the quality of nursing care and patient safety. It should be noted that some factors affecting MNCs are beyond the authority and control of nurses, so it is necessary to develop committees consisting of all hospital stakeholders to correct the factors affecting the missing nursing care. Further studies on the types and factors affecting missed nursing care and services in other countries will lead to the application of healthcare providers' experiences, including nurses, other health workers on the front lines of dealing with COVID-19, patients involved, and their families.

### 5.1. Limitations

Due to the qualitative nature of the study, the possibility that participants in the study did not remember or did not tend to express missed care and the factors shaping it was their fear of reprimand and punishing managers, officials, and other stakeholders due to the MCN report. Even though qualitative studies show the participants' in-depth experiences, they may suffer from the non-generalizability of the results. Moreover, due to the current pandemic, it was impossible to observe the care process and conduct focus groups to generate data.

### 5.2. Future studies

It is suggested that future research examine the frequency of MNC during this pandemic, as well as its possible consequences for patients and nurses.

## Data availability statement

The raw data supporting the conclusions of this article will be made available by the authors, without undue reservation.

## Ethics statement

This study was approved by the Ethics Committee of the Baqiyatallah University of Medical Sciences, Tehran, Iran, with the code: IR.BMSU.REC.1399.518 on 2 January 2020. The Declaration of Helsinki, including obtaining written and oral consent from all research participants to record their interviews and voluntary participation in the study, was observed. Participants were ensured of the data confidentiality and the right to withdraw from the study at any stage. In addition, all methods were performed in accordance with the relevant guidelines and regulations. A summary of the factors related to missed nursing care was reported to the care quality improvement committee of these three hospitals.

## Author contributions

AS, MR, and SB: study design. FK and ME: data collection. AS and RJ: data analysis. AS and SB: study supervision. AS, MR, SB, RJ, HA, and ME: manuscript writing. SB, MR, AS, HA, and FK: critical revisions for important intellectual content. All authors read and approved the final manuscript.
